# Characterization of Phytoconstituents from Alcoholic Extracts of Four Woody Species and Their Potential Uses for Management of Six *Fusarium oxysporum* Isolates Identified from Some Plant Hosts

**DOI:** 10.3390/plants10071325

**Published:** 2021-06-29

**Authors:** Mohamed Z. M. Salem, Abeer A. Mohamed, Hayssam M. Ali, Dunia A. Al Farraj

**Affiliations:** 1 Forestry and Wood Technology Department, Faculty of Agriculture (El-Shatby), Alexandria University, Alexandria 21545, Egypt; zidan_forest@yahoo.com; 2 Plant Pathology Institute, Agriculture Research Center (ARC), Alexandria 21616, Egypt; abeer_pcr@yahoo.com; 3 Botany and Microbiology Department, College of Science, King Saud University, P.O. Box 2455, Riyadh 11451, Saudi Arabia; dfarraj@ksu.edu.sa

**Keywords:** plant extracts, *Fusarium oxysporum* isolates, *Acacia saligna*, *Conium maculatum*, *Schinus terebinthifolius*, *Ficus eriobotryoides*, antifungal, antioxidant, HPLC

## Abstract

Background: Trees are good sources of bioactive compounds as antifungal and antioxidant activities. Methods: Management of six molecularly identified *Fusarium oxysporum* isolates (F. oxy 1, F. oxy 2, F. oxy 3, F. oxy 4, F. oxy 5 and F. oxy 6, under the accession numbers MW854648, MW854649, MW854650, MW854651, and MW854652, respectively) was assayed using four extracts from *Conium maculatum* leaves, *Acacia saligna* bark, *Schinus terebinthifolius* wood and *Ficus eriobotryoides* leaves. All the extracts were analyzed using HPLC-VWD for phenolic and flavonoid compounds and the antioxidant activity was evaluated using 2,2-diphenyl-1-picrylhydrazyl (DPPH) free radical scavenging and *β*-carotene-linoleic acid (BCB) bleaching assays. Results: In mg/kg extract, the highest amounts of polyphenolic compounds *p*-hydroxy benzoic, benzoic, gallic, and rosmarinic acids, with 444.37, 342.16, 311.32 and 117.87, respectively, were observed in *C. maculatum* leaf extract; gallic and benzoic acids with 2551.02, 1580.32, respectively, in *A. saligna* bark extract; quinol, naringenin, rutin, catechol, and benzoic acid with 2530.22, 1224.904, 798.29, 732.28, and 697.73, respectively, in *S. terebinthifolius* wood extract; and rutin, *o*-coumaric acid, *p*-hydroxy benzoic acid, resveratrol, and rosmarinic acid with 9168.03, 2016.93, 1009.20, 1156.99, and 574.907, respectively, in *F. eriobotryoides* leaf extract. At the extract concentration of 1250 mg/L, the antifungal activity against the growth of *F. oxysporum* strains showed that *A. saligna* bark followed by *C. maculatum* leaf extracts had the highest inhibition percentage of fungal growth (IPFG%) against F. oxy 1 with 80% and 79.5%, F. oxy 2 with 86.44% and 78.9%, F. oxy 3 with 86.4% and 84.2%, F. oxy 4 with 84.2, and 82.1%, F. oxy 5 with 88.4% and 86.9%, and F. oxy 6 with 88.9, and 87.1%, respectively. For the antioxidant activity, ethanolic extract from *C. maculatum* leaves showed the lowest concentration that inhibited 50% of DPPH free radical (3.4 μg/mL). Additionally, the same extract observed the lowest concentration (4.5 μg/mL) that inhibited BCB bleaching. Conclusions: Extracts from *A. saligna* bark and *C. maculatum* leaves are considered potential candidates against the growth of *F. oxysporum* isolates—a wilt pathogen—and *C. maculatum* leaf as a potent antioxidant agent.

## 1. Introduction

Trees and shrubs produce a broad range of bioactive compounds called secondary metabolism. These compounds have a long range of different effects as antimicrobials, antioxidants or insecticidal properties dependent on plant species and the type of bioactive compounds [1,2,3,4,5,6,7,8]. Medicinal and aromatic plants are often characterized as medicinal and poisonous depending on the presence of bioactive chemicals such as simple phenols, phenolic acids and flavonoid compounds [3,9,10]. However, in the literature regarding the bioactivity of flavonoids and polyphenols on antifungal activity, some results found that flavonoids were not associated with antifungal activity [11,12], while other works reported that the inhibition of fungal growth was mainly due to flavonoids [13,14].

*Conium maculatum* L., an umbelliferous weed, is known worldwide for its acute toxicity to humans and domestic animals [15]. Flavones (apigenin, luteolin, chrysoeriol), flavonols (kaempferol, quercetin, isorhamnetin), and anthocyanidins (cyanidin) have been detected in *C. maculatum* [16,17,18]. Other compounds furanocoumarins (psoralen, xanthotoxin and bergapten) were isolated from *C. maculatum* [19]. In addition, furocoumarins, polyines, prenylated coumarins and elemicin were isolated from root dichloromethane extract of *C. maculatum* [20]. Coniine (eight times more toxic than *γ*-coniceine) and *γ*-coniceine are the most abundant alkaloids with chronic toxicity found in *C. maculatum* extracts [21]. The leaf essential oil has only observed potential antifungal activity against *Aspergillus parasiticus* [22]. *C. maculatum* leaf extract exhibited maximum inhibition (100%) of *Verticillium fungicola* mycellial growth at a 1.5% concentration [23]. Meanwhile, in the study of Yanar et al. [24], *C. maculatum* leaf extract did not show any activity against the mycelial growth of *Alternaria solani*. *C. maculatum* showed significant relative antibacterial activity against *Staphylococcus aureus, Escherichia coli, Klebsiella pneumonia, Proteus vulgaris* and *Pseudomonas aeruginosa* [25]. In addition, the extract from *C. maculatum* as an herb presented good antibacterial activity against *S. aureus*, *B. subtilis*, *P. aeruginosa*, and *E. coli*, except against *C. albicans* according to measured MIC values [26]. 

*Acacia saligna* (Labill.) H. L.Wendl. is considered a fast-growing tree [27], and its extracts from different parts have shown some biologically active compounds as well antioxidant [3,28,29]. *Melia azedarach* wood treated with *A. saligna* flower extract showed good inhibition to *Penicillium chrysogenum* and moderate activity against *Fusarium culmorum* and *Rhizoctonia solani* but weak activity was reported [3]. Ethyl acetate extract of leaves was more effective as an antimicrobial than methanolic and water extracts [28]. The *A. cyanophylla* leaf ethanol extract showed potent antifungal activity against some species of *Aspergillus* [30].

*Schinus terebinthifolius* Raddi belongs to the family Anacardiaceae, and is a medicinal plant widely used for the treatment of various diseases as well for its own antimicrobial bioactive compounds [31,32,33,34]. Stem bark extract, which contains catechin, tannins, terpenes, flavonoids, and saponins, has shown a topical anti-inflammatory agent with potential antioxidant properties related to flavonoids [35]. Naringenin and gallic acid were identified in fruit extract with potent antioxidants and inhibit oxidative stress [36].

*Ficus* comprises about 800 species including shrubs, woody trees, and vines in the family Moraceae [37]. Extracts from different parts of *Ficus* species showed the presence of phenolic and flavonoid compounds with bioactivity properties such as antioxidant, antibacterial, antifungal and antiviral [38,39,40,41]. To the best of our knowledge, there are no studies in the literature considering the identification and characterization of the phenolic and flavonoid compounds as well other phytochemicals from *Ficus eriobotryoides* extracts.

Phytopathogenic fungi are posing major problems in agriculture. *Fusarium oxysporum* is a devastating wilt pathogen on almost 150 plant species. Fusarium with its toxic fumonisin mycotoxins has been shown to cause maize ear rot disease by contaminating its grains, which are major problems in pre- or post-harvest losses [42,43]. *F. oxysporum* is a causal pathogen for Panama wilt disease in *Musa paradisiaca* [44]. *F. oxysporum* is capable of causing vascular wilt, root rot and damping off diseases in over one hundred agronomically important plant species [45,46,47,48]. This pathogen is a soil-borne fungus and can survive in soil for more than ten years [49,50]. The plant extracts containing anti-fungal compounds have been gaining importance over the last three decades against a wide range of plant pathogenic microbes [51,52,53]. 

Trees and shrubs are renewable sources for raw materials, rich in valuable bioactive compounds including phenolic and flavonoid compounds [54,55]. The antioxidant activities of plant-derived phenolic compounds measured by 1,1-diphenyl-2-picrylhydrazyl (DPPH) free radical scavenging and beta-carotene bleaching (BCB) methods have been studied from extracts from several parts of trees like fruits, leaves, bark, seeds, flowers, and roots [56,57,58,59,60,61,62,63,64,65,66]. The antioxidant activity was well-correlated with the concentrations of phenolic, flavonoid and tannin contents [67,68]. Thus, in the present study, the plant extracts are tested for the inhibitory effect on the growth of the *F. oxysporum* pathogen.

The aim of the present study was to evaluate the biological activity of ethanol extracts four extracts of four plant species to control the wilt pathogen—*Fusarium oxysporum*. Phenolic and flavonoid compounds were also identified using HPLC-VWD, and the antioxidant activity was also reported.

## 2. Materials and Methods

### 2.1. Extraction of Plant Materials

Leaves of *Conium maculatum* L. were collected from the Garden of the Faculty of Agriculture, Alexandria University, Alexandria, Egypt, while *Acacia saligna* (Labill.) H.L.Wendl. (bark), *Schinus terebinthifolius* Raddi wood and *Ficus eriobotryoides* leaves collected from Antoniadis Gardens, Alexandria, Egypt, during June 2019, were used in the present study [8,27]. All the plant materials were identified by coauthor Dr. Mohamed Z.M. Salem at the Department of Forestry and Wood Technology, Faculty of Agriculture, Alexandria University. The plant materials were air-dried at room temperature until each of them could be transferred to powder using a small laboratory mill. After obtaining the powdered of all materials, 50 g from each plant material was extracted by soaking method [69,70], in 80% ethanol (150 mL) for one week, and then filtrated through cotton plug followed by filter paper (Whatman no. 1). The extracts were concentrated with evaporating the solvent using a rotary evaporator and poured in Petri dishes to complete the dryness. Three replicates for each extract were carried out. The afforded quantities of extracts were 4.45 ± 0.57, 9.57 ± 0.51, 7.06 ± 0.58, and 5.5 ± 0.81 g/100 g dry weigh from *C. maculatum* leaves, *A. saligna* bark, *S. terebinthifolius* wood, and *F. eriobotryoides* leaves, respectively. After that, the extracts were separately prepared in a stock solution of 200 mL as dissolved in 10% dimethyl sulfoxide (DMSO) and the following concentrations 500, 750, 1000 and 1250 mg/L were prepared. 

### 2.2. Phenolic and Flavonoid Compositions of Plant Extracts by HPLC Analysis

The phytochemical compounds of the ethanolic extracts from *C. maculatum* (leaves), *A. saligna* (bark), *S. terebinthifolius* wood and *F. eriobotryoides* (leaves) were injected and analyzed for their phytochemicals using An Agilent 1260 Infinity HPLC Series (Agilent, Santa Clara, CA, USA), equipped with a Quaternary pump and a Zorbax Eclipse plus C18 column (100 mm × 4.6 mm i.d.) (Agilent Technologies, Santa Clara, CA, USA) [3,9,71,72,73], with the injection volume of 20 μL and operated at 30 °C with the following ternary linear elution gradient;

(A) HPLC grade water 0.2% H_3_PO_4_ (*v*/*v*)
(B) methanol
(C) acetonitrile

Standard HPLC-grade phenolic and flavonoid compounds pyrogallol, quinol, gallic acid, catechol, *p*-hydroxy benzoic acid, chlorogenic acid, vanillic acid, caffeic acid, syringic acid, vanillin, *p*-coumaric acid, ferulic acid, benzoic acid, rutin, ellagic acid, *o*-coumaric acid, salicylic acid, resveratrol, cinnamic acid, myricetin, quercetin, rosmarinic acid, naringenin and kaempferol as well as caffeine, were used for the HPLC analysis. The detection was set at 284 nm to identify the phenolic compounds.

### 2.3. Antifungal Activity and Minimum Inhibitory Concentration (MIC) Assays of Four Plant Extracts

The antifungal activity of four plant extracts was assessed against six fungal isolates of *Fusarium oxysporum* F. oxy 1, F. oxy 2, F. oxy 3, F. oxy 4, F. oxy 5 and F. oxy 6, collected from different plant hosts of Peas (*Pisum sativum* L.), Zucchini (*Cucurbita pepo* L.), Egyptian Rice (*Oryza sativa* L.), Pepper (*Capsicum annuum* L.), Cape gooseberry (*Physalis peruviana* L.), and Bean (*vicia faba* L.) with their sequencing ITS regions submitted and registered to GenBank under the accession numbers MW854648, MW854649, MW854650, MW854651, MW854652, and MW854653, respectively. The plant extracts were prepared as mentioned above at the concentrations of 500, 750, 1000 and 1250 mg/L [74]. Carbendazim (reference chemical fungicide) prepared at concentrations of 200 mg/L were assessed using the broth dilution method according to Clinical and Laboratory Standards Institute (CLSI) [75]. *F. oxysporum* isolates were cultivated on a PDA medium. Then, a single 0.5 cm culture disk was taken from actively growing cultures and placed in the middle of the Petri dishes were with the different concentrations of plant extracts. The plates were incubated for 6 days at 28 °C, and three replications were used for each isolate [53,76,77]. The fungal inhibition percentage was calculated with the formula of inhibition percentage of fungal growth (IPFG) (%) = [DC-DT/DC] × 100, where DC and DT are the average diameters (mm) of fungal colonies under the control and experimental treatments, respectively. Three replicates were carried out for all of the treatments [52]. The minimum inhibitory concentrations (MIC) of the plant extracts prepared at concentrations of 64 to 1250 mg/L were assessed according to CLSI [75].

### 2.4. Antioxidant Activity of the Extracts

Free radical scavenging activity of the obtained four extracts was measured using 2,2-diphenyl-1-picrylhydrazyl (DPPH) method (absorbance at 517 nm), along with the β-carotene-linoleic acid (BCB) bleaching assay [71,78,79]. The DPPH is a stable free radical alcohol soluble and the assay is based on its scavenging by the active principles of the extracts, while BCB assay is based on the bleaching inhibition of this system by the extract biocompounds. The concentration of extract or the references compounds ascorbic acid (AA) and butylated hydroxyl toluene (BHT)) responsible for 50% of inhibition of DPPH radical or BCB bleaching inhibition after 24 h of incubation was determined [80,81,82].

### 2.5. Statistical Analysis 

The results of the percentages of the fungal linear inhibition of six isolates of *Fusarium oxysporuma* as affected by four concentrations (500, 750, 1000 and 1250 mg/L) of the ethanol extract of *C. maculatum* leaves, *A. saligna* bark, *S. terebinthifolius* wood and *Ficus eriobotryoides* leaves were statistically analyzed with two-way analysis of variance (ANOVA) using SAS software (SAS Institute, Release 8.02, Cary, North Carolina State University, Raleigh, NC, USA) [83]. The means were compared against the control treatment according to Duncan’s Multiple Range Test at a 0.05 level of probability.

## 3. Results

### 3.1. Phytochemical Analysis of Extracts by HPLC

**Figure 1:**
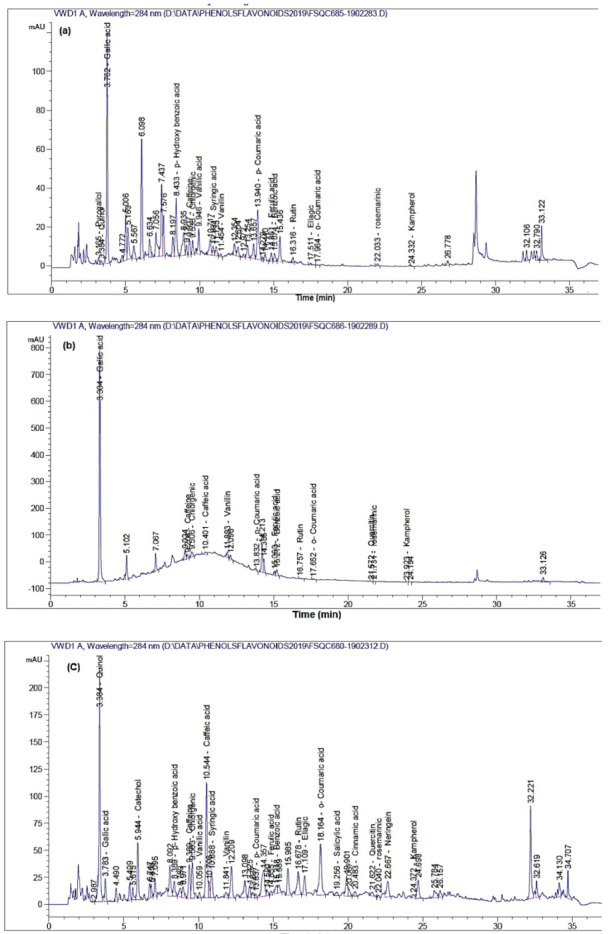

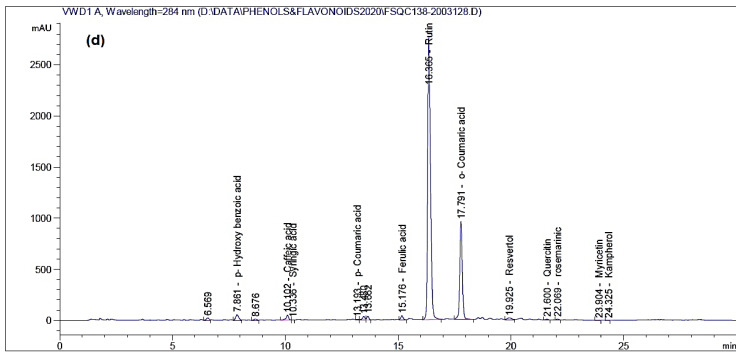
HPLC analysis of extracts from (**a**) *Conium maculatum* leaves*;* (**b**) *Acacia saligna* bark; (**c**) *Schinus terebinthifolius* wood; and (**d**) *Ficus eriobotryoides* leaves.

Table 1 presents the chemical compounds of the phenolic and flavonoid compounds as well as caffeine identified in the 80% ethanolic extracts from *Conium maculatum* leaves, *Acacia saligna* bark, *Schinus terebinthifolius* wood, and *Ficus eriobotryoides* leaves. Figure 1 shows the HPLC chromatograms of the identified compounds from studied extracts. The highest amounts (mg/kg extract) of chemical compounds *p*-hydroxy benzoic acid (444.37), benzoic acid (342.16), gallic acid (311.32), rosmarinic acid (117.87), vanillic acid (95.21) and *p*-coumaric acid (81.86) were observed in *C. maculatum* leaf extract. *A. saligna* bark extract showed the presence of gallic acid (2551.02), benzoic acid (1580.32), caffeine (106.73), and chlorogenic acid (103.50) followed by vanillin (69.46), caffeic acid (53.55), rosmarinic acid (49.57) and ferulic acid (42.17) as main compounds in mg/kg extract. The compounds quinol (2530.22), naringenin (1224.904), rutin (798.29), catechol (732.28), benzoic acid (697.73), quercetin (315.44), caffeic acid (302.27), caffeine (267.62), *p*-hydroxy benzoic acid (233.27), rosmarinic acid (187.66), chlorogenic acid (174.65), kaempferol (175.06) and *o*-coumaric acid (139.04) were observed as the highest amounts (mg/kg extract) identified in *S. terebinthifolius* wood extract. In the ethanol extract of *F. eriobotryoides* leaves, the highest peaks (mg/kg extract) observed were rutin (9168.03), *o*-coumaric acid (2016.93), *p*-hydroxy benzoic acid (1009.20), resveratrol (1156.99), and rosmarinic acid (574.907).

### 3.2. Antifungal Activity of Extracts

**Figure 2:**
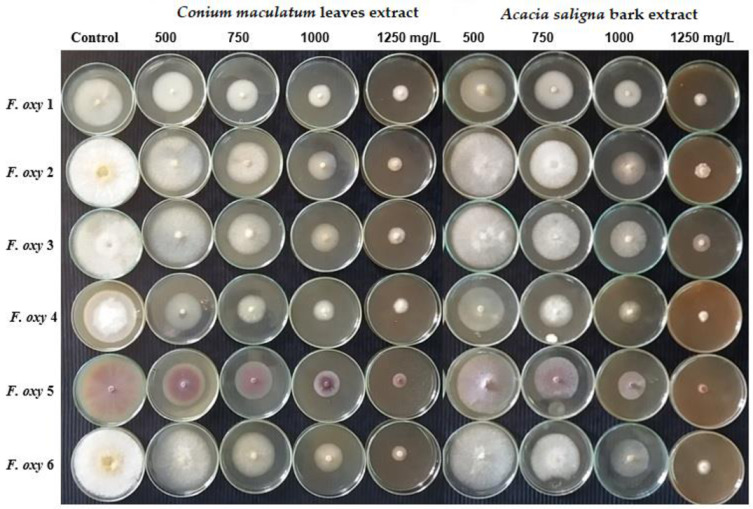

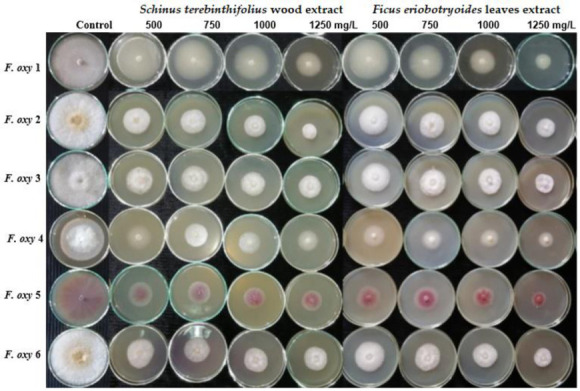
Visual observations of the antifungal activity of extracts from *Conium maculatum* leaves, *Acacia saligna* bark, *Schinus terebinthifolius* wood and *Ficus eriobotryoides* leaves against six isolates of *F. oxysporum*.

Figure 2 shows the visual observation of the activity of four plant extracts (*C. maculatum* leaves, *A. saligna* bark, *S. terebinthifolius* wood and *F. eriobotryoides* leaves against six isolates of *F. oxysporum*. It can be seen that with the increase in the extract’s concentration, the mycelial inhibition percentage of fungi is increased. 

Table 2 presents the antifungal activity of extracts against the growth of six isolates of *F. oxysporum.* The highest inhibition percentage of fungal growth (IPFG%) against the growth of F. oxy 1 was observed with extracts from *A. saligna* bark followed by *C. maculatum* leaves at 1250 mg/L with IPFG of 80%, and 79.5%, respectively, while *F. eriobotryoides* leaf extract showed good activity with IPFG of 73.1% at 1250 mg/L. However, these values are lower than the value from carbendazim (88.89%). Extract from *A. saligna* bark showed the potent antifungal activity against isolate F. oxy 2 with IPFG of 86.4% at 1250 mg/L, which higher than the values from carbendazim (85.2%). Furthermore, extracts from *C. maculatum* leaves, *S. terebinthifolius* wood and *F. eriobotryoides* leaves showed good activity against the growth of F. oxy 2 with IPFG values of 78.9, 73.5, and 66.6%, respectively, at the concentration of 1250 mg/L. Extracts from *A. saligna* bark (IPFG 86.4%), and *C. maculatum* leaves (IPFG 84.2%) showed the highest activity against the growth of F. oxy 3, which higher than the IPFG from carbendazim (79.2%). In addition, *F. eriobotryoides* leaf extract at the concentration of 1250 mg/L observed IPFG value of 86.4% against F. oxy 3. At concentration of 1250 mg/L, extract from *A. saligna* bark, *C. maculatum* leaves, and *F. eriobotryoides* leaves showed the highest activity against the growth of F. oxy 4 with IPFG values of 84.2, 82.1, and 76.6, respectively, and were higher than the values from carbendazim (75.8%). Extracts from *A. saligna* bark and *C. maculatum* leaves observed the highest activity against F. oxy 5 with IPFG values of 88.4% and 86.9%, respectively, and those values were higher than the reported from carbendazim (84.81%). In addition, *F. eriobotryoides* leaves extract at the concentration of 1250 mg/L showed good activity against F. oxy 5 with an IPFG value of 82.9%. Extracts from *A. saligna* bark and *C. maculatum* leaves showed a significant effect against F. oxy 6 with values of IPFG 88.9, and 87.1%, respectively, and these values were highest than the value of carbendazim (82.6%). The MIC values (mg/L) measured against the growth of six isolates from *F. oxysporum* are shown in Table 3. The range of these values were 32–125, 64–125, 125–250, and 125–250 mg/L, as the extracts from *C. maculatum*, *A. saligna*, *S. terebinthifolius* and *F. eriobotryoides*, respectively, were measured. Nevertheless, these values were lower than the reported from carbendazim (5–10 mg/L).

### 3.3. Antioxidant Activity of Extracts 

Table 4 presents the antioxidant activity of extracts from *C. maculatum* leaves, *A. saligna* bark, *S. terebinthifolius* wood and *F. eriobotryoides* leaves compared with those reported from the standards ascorbic acid (AA) and butylated hydroxyl toluene (BHT) as measured by 2,2-Diphenyl-1-picrylhydrazyl (DPPH) free radical scavenges and β-Carotene-linoleic acid bleaching (BCB) assays. The lowest concentrations that inhibited 50% of DPPH free radicals were 3.4 μg/mL (*C. maculatum* leaves) and 5.12 μg/mL (*S. terebinthifolius* fruits) where they were lower than the value from AA (7.66 μg/mL) but higher than from BHT (2.4 μg/mL). Comparing with the other method, BCB, the lower values were reported as *C. maculatum* extract (4.5 μg/mL) was tested, which was lower than the value reported from AA (5.12 μg/mL) and higher than as found be BHT (2.78 μg/mL). It can be observed that the extract from *A. saligna* bark had weakened antioxidant activity as measured by DPPH and BCB methods. 

## 4. Discussion

The results of the present work show that the extracts of *C. maculatum* leaves, *A. saligna* bark, *S. terebinthifolius* wood and *F. eriobotryoides* leaves possessed a remarkable and potential antifungal activity against the six *F. oxysporum* isolates as well as antioxidant properties. These activities could be related to the presence of the identified several phenolic and flavonoid compounds in their extracts. 

*p*-Hydroxy benzoic acid, benzoic acid, gallic acid, rosmarinic acid, vanillic acid and p-coumaric acid were observed as the abundant compounds in *C. maculatum* leaf ethanolic extract. Previously, total phenolic compounds were presented in *C. maculatum* 33.28 mg GAE/g DW [84]. Coumarins, umbelliferone and scopoletin compounds isolated from *C. maculatum* extract showed inhibitory effects on *Alternaria*, and *Bipolaris* species spore germination, which were greater than those of xanthotoxin, furanocoumarins, bergapten and angelicin [85]. The tested furanocoumarins were most effective for inhibiting mycelial growth of *Fusarium* spp. than *Alternaria* and *Bipolaris* [85]. Leaf extract of *C. maculatum* showed weak activity against *Phytophthora infestans* [86]. At the concentration of 50%, *C. maculatum* roots ethanolic extract showed the maximum inhibition of mycelia growth and conidial germination of the *Fusarium pallidoroseum* [87].

In the present study, bark extract from *Acacia saligna* showed the presence of gallic acid, benzoic acid, caffeine, chlorogenic acid, vanillin, caffeic acid, rosmarinic acid and ferulic acid as main compounds. Polyphenolic and tannins compounds are the most abundant compounds in leaves, fruits, stems, pods, petiole, and roots of *Acacia* [88,89]. Previous work showed that benzoic acid, caffeine, *o*-coumaric acid, naringenin, quercetin, and kaempferol were identified as the main compounds from water extract of *A. saligna* flower analyzed using HPLC, but in total, the extract showed weak antioxidant activity as measured by the DPPH method [3]. *A. saligna* leaf extracts qualitatively showed the presence of polyphenolic compounds, e.g., quercetin, quercitrin, apigenin, apigenin-7-glucoside, myricetin 3-*O*-glucoside, astragalin, gallic acid luteolin, myricetin, myricitrin, 7-galloylcatechin, (+)-catechin and kaempferol [28,90,91]. Myricetin-3-*O*-rhamnoside (C7-*O*-C7) myricetin-3-*O*-rhamnoside was isolated from leaves while myricetin-3-O-*α*-L-rhamnoside and quercetin-3-*O*-*α*-*L*-rhamnoside were isolated from leaves and flowers of *A. saligna* [92]. 

Acacia extracts were observed to exhibit potent bioactivity against a wide range of fungal species including Pythium aphanidermatum, Alternaria brassicae, Rhizoctonia solani, Microsporum gypseum, Epidermophyton floccosum, Trichophyton rubrum and Fusarium oxysporum ciceris, F. culmorum, Candida albicans and Penicillium chrysogenum [3,93,94,95,96,97]. In the present study, the illustration of the fungicidal bioactivity of A. saligna bark extract against six isolates of F. oxysporum is shown for the first time. Recently, strong antifungal activity was observed with methanolic extract of leaves, which were associated with specific polyphenols gallic acid, quercetin 3-glucuronide, rutoside, hyperoside, and p-coumaric acid [98]. Leaf ethanol extract of A. saligna showed remarkable antifungal activity against Aspergillus flavus, A. fumigatus, A. niger, and Candida albicans, where phenolic acid gallic, protocatechuic, chlorogenic, p-hydroxy benzoic, p-coumaric, syringic, vanillic and salicylic were reported as main compounds [29]. From other species of Acacia, methanol extract from Acacia ampliceps bark showed significant to moderate inhibition against Trichoderma spp., Rhizopus and Acremonium spp. and less activity against Aspergillus niger [99]. Fruits and bark ethyl acetate extracts of A. nilotica (L.) Willd. ex Del subsp. nilotica, tomentosa and astringens showed the highest molluscicidal activity against Bulinus truncatus and Biomphalaria pfeifferi. The activity was mainly due to (-)-epigallocatechin-7-gallate and (-)-epigallocatechin-5,7-digallate or (-)-epigallocatechin derivatives [100]. Quercetin 3-O-(4’-O-acetyl)-rhamnopyranoside and ferulic acid was isolated from leaves and bark extract from A. arabica [101]. The whole plant extract from A. plicosepalus showed the presence of rutin [102].

Wood extract of *S. terebinthifolius* showed the present quinol, naringenin, rutin, catechol, benzoic acid, quercetin, and caffeic acid as the main abundant polyphenolic compounds. Gallic acid, methyl and ethyl gallates, (+)-catechin, myricetin, kaempferol, quercitrin, afzelin, and myricetrin were isolated from the leaf extract with cytotoxic and antiradical activities [103]. naringenin with gallic acid were identified in fruits extract from *S. terebinthifolius* [36]. Wood extract showed the presence of fatty acids in form of methyl esters such as myristic, 14-pentadecenooic acid, and pentadecanoic acid [104]. Phenolic compounds ferulic acid, caffeic acid, romarinic acid, chlorogenic acid, gallic acid and quercetin were identified in *S. terebinthifolius* extracts [105].

Phenolic compound of *S. terebinthifolius* might be useful in the control of *Paracoccidioides brasiliensis*, the pathogenic fungi [106]. Gallotannins, gallic acid and flavonoids were isolated from fruit of *S. terebinthifolius* with potential antibacterial activity [107]. Gallic acid and its derivatives have been isolated from leaves and fruits of *S. terebinthifolius* [108,109]. Additionally, leaf extract from *S. terebinthifolius* showed the presence of two gallic acid derivatives, methyl gallate and 1,2,3,4,6-penta-*O*-galloyl-*β*-glucopyranoside, and four flavonoids (robustaflavone, quercetin, quercetrin, and luteolin), where they exhibited considerable antioxidant activity [110]. Numerous bioactive compounds were identified from the aerial parts extract of *S. terebinthifolius* such as coumarins, 2,8-dihydroxyadenine, gallic acid and tannins [111,112].

In the present study and for the first time, we identified the polyphenolic compounds from *Ficus eriobotryoides* leaves, where rutin, o-coumaric acid, *p*-hydroxy benzoic acid, resveratrol, and rosmarinic acid were identified as the main compounds in the ethanol extract. Phenolic compounds such as furanocoumarins (psoralen and bergapten), ferulic acid, gallic acid, chlorogenic acid, and flavonoids like rutin identified from some *Ficus* plants have been recognized for their pharmacological properties [41,113,114,115]. The strong antioxidant and antibacterial activities of *F. microcarpa* bark extract have been attributed to its high level of phenolic compounds such as catechol, vanillin, syringaldehyde, *p*-propylphenol, *p*-vinylguaiacol, and syringol [116]. Rutin, and chlorogenic acid, present in *F. carica*, and *F. elastica* extracts have been promised as potent antioxidant activity [117].

Phenolic and flavonoid compounds found in plants with different quantities depending on the plant part and the extraction process have great effects as antimicrobials and antioxidants [3,71,118]. Dihydroquercetin isolated from barley showed to suppress the growth of *Fusarium* spp. [119], while naringenin and its derivatives were displayed potential antimicrobial activities [120]. The methanol extract with its main compound rutin extracted from peels of *Musa paradisiaca* showed potential wood-biofungicide against the growth of *Fusarium culmorum* and *Rhizoctonia solani* [9]. Flower extract of *A. saligna* flower extract with its main phenolic and flavonoid compounds (*o*-coumaric acid, benzoic acid, quercetin, naringenin, and kaempferol) showed good antifungal activity against *Penicillium chrysogenum* [3]. Rutin from *Polygala paniculata* possessed good activity against *Sporothrix schenckii* and *Cryptococcus gattii* [121], while the extract from *Phaleria macrocarpa* fruit showed the presence of myricetin, naringin, and rutin, which could responsible for the bioactivity [122,123]. Quercetin, which was identified in *S. terebinthifolius* wood and *F. eriobotryoides* leaves, has shown antifungal and antioxidant activities [124]. Three flavonoids and two esters of gallic acid isolated from *S. terebinthifolius* leaves were observed for their antiradical potential [103].

## 5. Conclusions

This study provides the potential use of four extracts from *Conium maculatum* leaves, *Acacia saligna* bark, *Schinus terebinthifolius* wood and *Ficus eriobotryoides* leaves for the antifungal and antioxidant properties. Phytochemical investigations of the ethanolic extracts identified several phenolic and flavonoid compounds, where the most abundant compounds were *p*-hydroxy benzoic acid, benzoic acid, and gallic acid in *C. maculatum* leaf, gallic and benzoic acids in *A. saligna* bark, quinol, naringenin, rutin, catechol, benzoic acid, and quercetin in *S. terebinthifolius* wood, and rutin, *o*-coumaric acid, *p*-hydroxy benzoic acid, resveratrol, and rosmarinic acid in *F. eriobotryoides* leaves. The extracts showed promising antifungal and antioxidant properties. Extracts from *A. saligna* and *C. maculatum* showed the highest activity against all the studied six isolates from *F. oxysporum*. Among the four extracts, *C. maculatum* leaf extract showed promising antioxidant activity compared to standard antioxidant compounds. Therefore, the phenolic and flavonoid compounds as well as caffeine present in the four plants were identified as a promising natural source to control and manage the growth of *Fusarium oxysporum* isolates as well as for antioxidant activity.

## Figures and Tables

**Table 1:** Phytochemical compounds of extracts by HPLC analysis.

Compound	Amount (mg/kg Extract)							
	*C. maculatum* Leaves		*A. saligna* Bark		*S. terebinthifolius* Wood		*F. eriobotryoides* Leaves	
	RT *	Amount	RT	Amount	RT	Amount	RT	Amount
Pyrogallol	3.165	20.57	-	ND	-	ND	-	ND
Quinol	3.384	23.27	-	ND	3.384	2530.22	-	ND
Gallic acid	3.762	311.32	3.304	2551.02	3.783	67.87	-	ND
Catechol	-	ND	-	ND	5.944	732.28	-	ND
*p*-Hydroxy benzoic acid	8.433	444.37	-	ND	8.389	233.27	7.861	1009.20
Caffeine	9.327	50.63	9.204	106.73	9.389	267.62	-	ND
Chlorogenic acid	9.536	30.99	9.506	103.50	9.583	174.65	-	ND
Vanillic acid	9.946	95.21	-	ND	10.059	33.97	-	ND
Caffeic acid	-	ND	10.401	53.55	10.544	302.27	10.102	157.48
Syringic acid	10.931	18.87	-	ND	10.888	109.28	10.336	5.90
Vanillin	11.454	5.71	11.883	69.46	11.841	23.92	-	ND
*p*-Coumaric acid	13.940	81.86	13.832	8.27	13.837	3.58	13.193	0.0935
Ferulic acid	14.851	15.15	15.060	42.17	14.855	12.94	15.176	145.45
Benzoic acid	15.074	342.16	15.212	1580.32	15.218	697.73	-	ND
Rutin	16.316	19.93	16.757	16.15	16.678	798.29	16.365	9168.03
Ellagic acid	17.511	5.74	-	ND	17.109	116.32		ND
*o*-Coumaric acid	17.964	5.82	17.652	11.44	18.164	139.04	17.791	2016.93
Salicylic acid	-	ND	-	ND	19.256	78.35	-	ND
Resveratrol	-	ND	-	ND	-	ND	19.925	1156.99
Cinnamic acid	-	ND	-	ND	20.483	7.16	-	ND
Quercetin	-	ND	21.572	37.36	21.622	315.44	21.600	314.85
Rosmarinic acid	22.033	117.87	21.731	49.57	22.040	187.66	22.069	574.907
Naringenin	-	ND	-	ND	22.667	1224.904	-	ND
Myricetin	-	ND	-	ND	-	ND	23.904	65.23
Kaempferol	24.332	16.41	23.923	10.73	24.372	175.06	24.325	10.95

*: RT: Retention time (min); ND: not detected

**Table 2:** Antifungal activity of plant extracts against six isolates of *F. oxysporum*.

Plant Extracts	Conc. (mg/L)	Inhibition Percentage of Fungal Growth (%)					
		F. oxy 1	F. oxy 2	F. oxy 3	F. oxy 4	F. oxy 5	F. oxy 6
Control ^a^	0	0.00	0.00	0.00	0.00	0.00	0.00
Positive control ^b^	200	88.9 ± 1.1	85.2 ± 0.6	79.2 ± 1.7	75.8 ± 1.1	84.8 ± 0.6	82.6 ± 0.6
*C. maculatum* leaves	500	27.9 ± 1.8	12.6 ± 1.1	13.7 ± 0.7	34.8 ± 1.4	40.2 ± 1	8.6 ± 1.7
	750	41.5 ± 3.4	48.4 ± 1.6	40.4 ± 0.7	44.5 ± 0.9	47.5 ± 0.7	30.4 ± 2.7
	1000	62.1 ± 2.6	57.1 ± 1.4	60.2 ± 1	68.7 ± 0.5	66.9 ± 1	58.2 ± 1.7
	1250	79.5 ± 0.3	78.8 ± 1	84.2 ± 1.4	82.1 ± 0.5	86.9 ± 0.7	87.1 ± 0.4
*A. saligna* bark	500	9.4 ± 0.5	11.3 ± 2	15.3 ± 1.7	9.4 ± 0.5	13.1 ± 0.4	6.2 ± 1.4
	750	27.3 ± 0.9	33.1 ± 0.4	31.7 ± 1.6	44.8 ± 1	29.7 ± 3.1	32.6 ± 0.6
	1000	49.4 ± 3.4	58.2 ± 1.6	46.6 ± 1.3	56.6 ± 1.9	62 ± 1.7	47.3 ± 0.6
	1250	80 ± 1.5	86.4 ± 0.4	86.4 ± 0.4	84.2 ± 2.1	88.4 ± 1.5	88.8 ± 0.8
*S. terebinthifolius* wood	500	12.6 ± 0.6	46.8 ± 1	53.5 ± 1	30 ± 0.7	49.4 ± 0.6	49.5 ± 1.7
	750	30 ± 3.1	52.8 ± 0.4	54.8 ± 1.4	50.7 ± 2.7	55.8 ± 0.3	54.6 ± 1.3
	1000	40 ± 0.6	59.3 ± 0.6	57.3 ± 0.6	60.5 ± 0.9	62.7 ± 0.9	60 ± 0.6
	1250	54.6 ± 1.3	73.5 ± 1	57.1 ± 0.4	61.8 ± 0.4	68.7 ± 0.6	60.2 ± 0.4
*F. eriobotryoides* leaves	500	30 ± 2.9	46.6 ± 0.6	41.1 ± 1	53.1 ± 0.7	54.8 ± 1.3	46.6 ± 0.6
	750	36.4 ± 3.1	52.8 ± 0.4	53.3 ± 0.6	61.3 ± 0.4	60.6 ± 1.9	56.4 ± 3.1
	1000	52.6 ± 0.6	58.4 ± 1.6	60.2 ± 1	67.94 ± 1.2	63.9 ± 1.6	59.3 ± 0.6
	1250	73.1 ± 1.9	66.6 ± 0.6	66.6 ± 0.6	76.6 ± 0.4	82.9 ± 1.6	71.5 ± 1.7
*p*-Value		**	**	**	**	**	**

^a^: Negative control (DMSO), ^b^: Positive control (Carbendazim); **: Highly significant effect at 0.01 level of probability.

**Table 3:** Minimum inhibitory concentrations (MICs) of the plant extracts and reference fungicide.

Plant Extracts	Minimum Inhibitory Concentration (MIC mg/L) against *F. oxysporum* Isolates					
	F. oxy 1	F. oxy 2	F. oxy 3	F. oxy 4	F. oxy 5	F. oxy 6
*Conium maculatum* leaves	125	125	64	32	64	64
*Acacia saligna* bark	125	125	64	64	64	64
*Schinus terebinthifolius* wood	125	250	250	125	250	250
*Ficus eriobotryoides* leaves	125	250	250	125	125	250
Carbendazim *	10	10	5	5	10	10

* Reference fungicide.

**Table 4:** Antioxidant activity of four extracts measured by DPPH and *β*-carotene-linoleic acid assays.

Extract	Concentration (μg/mL) *	
	DPPH	BCB
*Conium maculatum* leaves	3.4 ± 0.1 ^e^^**^	4.5 ± 0.1 *^d^*
*Acacia saligna* bark	10.2 ± 0.1 *^a^*	14.3 ± 0.1 *^a^*
*Schinus terebinthifolius* wood	5.12 ± 0.4 *^c^*	6 ± 0.12 *^b^*
*Ficus eriobotryoides* leaves	4.22 ± 0.12 *^d^*	6.07 ± 0.33 *^b^*
Positive controls		
AA	7.66 ± 0.5 *^b^*	5.12 ± 0.1 *^c^*
BHT	2.4 ± 0.2 *^f^*	2.78 ± 0.1 *^e^*

*: The lowest concentration that caused a 50% inhibition of free radical by DPPH method or by 50% BCB bleaching inhibition compared with control. AA: Ascorbic acid. BCB: β-Carotene-linoleic acid. BHT: Butylated hydroxyl toluene. DPPH: 2,2-Diphenyl-1-picrylhydrazyl. All the values are mean ± SD. SD: standard deviation. The lowest values are the most active. ** Means with the same superscript letter within the same column are not significantly different according to LSD (*p* < 0.05).

## Data Availability

Not applicable.
